# Neurocognitive health of older adults experiencing homelessness in Oakland, California

**DOI:** 10.3389/fneur.2022.905779

**Published:** 2022-07-22

**Authors:** Sandeepa Satya-Sriram Mullady, Stacy Castellanos, Lucia Lopez, Gloria Aguirre, John Weeks, Stephen King, Karen Valle, Collette Goode, Elena Tsoy, Katherine Possin, Bruce Miller, Margot Kushel, Serggio Lanata

**Affiliations:** ^1^Department of Neurology, Memory and Aging Center, UCSF Weill Institute for Neurosciences, University of California, San Francisco, San Francisco, CA, United States; ^2^Department of Internal Medicine, UCSF Center for Vulnerable Populations, University of California, San Francisco, San Francisco, CA, United States

**Keywords:** homelessness, underserved populations, social determinants of health, neurodegenerative disease, neurocognitive testing, dementia, neurocognitive disorders, neurological health

## Abstract

**Background and objectives:**

The homeless population in the US is aging. Cognitive impairment is prevalent in this population, yet little is known about the neurologic etiologies of such impairment. Addressing this gap in knowledge is important because homeless older adults with cognitive impairment due to neurodegenerative disease may need lifelong tailored support to obtain and maintain housing. In this study, we characterized the neurocognitive health of a sample of adults who experienced homelessness for the first time after age 50 using gold standard behavioral neurology examination practices.

**Methods:**

We conducted a descriptive cross-sectional study of older adults who first experienced homelessness after age 50. We recruited our sample purposively from an ongoing longitudinal cohort study of adults who were aged 50 and over and homeless when they entered the cohort. For this sub study, we enrolled a convenience sample from those who reported their first episode of homelessness after age 50. We did not exclude individuals based on history of substance use. Neurologists conducted a structured neurocognitive history intake, neurological examination, neuropsychological evaluation, and functional assessment between November 2020 and February 2021. We screened all participants for neurocognitive disorders using gold standard clinical research diagnostic criteria.

**Results:**

We evaluated 25 participants, most were men (76%) and Black (84%), with a median age of 61 years. The most common neurocognitive complaints included deficits in recent episodic memory (*n* = 15, 60%), executive functions (*n* = 13, 52%), and behavior/mood, with apathy being the most common complaint (*n* = 20, 80%). Neuropsychological testing revealed a high prevalence of socioemotional deficits (*n* = 20, 80%). Common neurological examination deficits included difficulties with coordination, such as impaired Luria task (*n* = 16, 64%), signs of distal peripheral neuropathy (*n* = 8, 32%), anosmia/hyposmia (*n* = 4, 21%), and signs of mild Parkinsonism (*n* = 5, 20%). The most common diagnoses were MCI (*n* = 7, 28%), bvFTD (*n* = 4, 16%), AD (*n* = 4, 16%), and DLB (*n* = 2, 8%).

**Discussion:**

Our findings suggest that neurocognitive concerns and examination deficits are common among older homeless adults. Specific neurocognitive disorders may be overrepresented in this population, particularly frontotemporal disorders. Longitudinal studies involving brain biomarkers are needed to characterize the neurocognitive health of this vulnerable population more precisely.

## Introduction

In the 1990s, ~11% of persons experiencing homelessness in San Francisco were 50 years of age and older, and by 2013, this figure had risen to 32% (1). Similar demographic trends exist in other metropolitan areas of the country, with the number of persons who are homeless and elderly (≥ 65 years) expected to triple nationally by 2030 (2). An increasing number of unhoused adults are becoming homeless for the first time after 50 years of age (1, 3). Among those 50 and older and homeless, 44% had never experienced homelessness prior to age 50.

Studying the brain health of older adults (≥ 50) and elderly (≥ 65) persons experiencing homelessness is important for two main reasons. First, the incidence and prevalence of different forms of age-related neurodegenerative disease of the brain (NDDB), such as Alzheimer disease (AD) and frontotemporal lobar degeneration (FTLD), is expected to rise as the general population ages (4). The early clinical signs and symptoms of some forms of NDDB, especially those that affect the anterior frontal and temporal lobes early in the illness (i.e., atypical presentations of AD and typical presentations of FTLD) lead to focal deficits in social cognition and socioemotional processing. Persons affected by NDDB often engage in behaviors that are harmful to their personal and social lives, intimate relationships, and work responsibilities (5, 6). We hypothesize that early changes in cognition caused by NDDB, particularly changes in social cognition, could precipitate homelessness in selected individuals, especially those who live alone, are minimally supported, or are socioeconomically vulnerable (7, 8). Second, most of the non-communicable health conditions that are disproportionately prevalent in homeless older adults are known risk factors for NDDB (9). There is a high prevalence of cognitive impairment and accelerated cognitive aging among older adult homeless persons (10–14), and at least 48% of homeless individuals with cognitive impairment exhibit signs of functional decline (15), yet little is known about the role that NDDB play in leading to cognitive impairment and functional decline in homeless older adults.

Studies on cognitive impairment in homeless adults generally rely on limited, brief assessments of cognition to evaluate participants (13) without incorporating more comprehensive neuropsychological, neurological, functional, and biomarker examinations that are currently considered gold standard practices in the evaluation of persons with suspected NDDB. Few studies have characterized cognitive impairment in this population using contemporary neurological approaches, including brain health biomarkers (16, 17). Such approaches are important as they may greatly inform prognosis of these older adults as well as guide the provision of person-centered supportive measures to help them obtain and maintain housing.

To study the intersection between NDDB and the experience of homelessness among older adults, we previously investigated relationships between homelessness and NDDB among an existing cohort of adult research participants with different forms of NDDB evaluated in the Memory and Aging Center (MAC) of the University of California San Francisco (UCSF) (8). We found that persons with anterior frontal and temporal neurocognitive deficits (i.e., persons with FTLD or the frontal variant of AD, for example) were highly represented among our homeless cohort, and most of these participants had become homeless in the setting of a fragile socioeconomic context.

In this study, we seek to expand this work by characterizing the neurocognitive health of a community-based cohort of older adults who experienced homelessness for the first time after the age of 50, employing gold standard neurological and neuropsychological examination approaches currently in use at the UCSF MAC.

## Methods

### Study overview

This study is a collaboration between the UCSF MAC Alzheimer Disease Research Center (ADRC) and the Health Outcomes in People Experiencing Homelessness in Older Middle agE (HOPE HOME) study. The UCSF Institutional Review Board approved the study.

### Participants and study design

We recruited participants from the HOPE HOME study, which used a venue-based sampling method to enroll 350 participants between 2013 and 2014, 100 additional participants between 2017 and 2018, and followed all participants at 6 months intervals since. At enrollment, participants needed to be age 50 and older (53 in second wave), homeless by the HEARTH criteria, and speak English. Participants remained in the study regardless of housing status at follow-up. HOPE HOME sampled from all overnight homeless shelters serving homeless adults in Oakland, CA, all free and low-cost meal programs serving three or more meals a week, and a random selection of homeless encampments and recycling programs. Staff sampled randomly within sites in order to reflect the population of interest (18–20). Staff obtained consent using a teach-back method and excluded individuals unable to give informed consent (21). We used multiple modalities to provide consent (written and oral) (22, 23). We assessed understanding by conducting a post-consent quiz that documents the participants' knowledge of critical elements of the informed consent (e.g., knowledge of components of the study, risks, voluntary nature of study, ability to withdraw, confidentiality, etc). We administered a post-exam quiz to assess understanding. For subjects who score less than perfect on the initial presentation, educational procedures were employed to raise their understanding to sufficient levels for them to make a meaningful choice about participating. Such procedures may include simple repetition of the relevant information in the consent form or more detailed explanations of items that the subject has difficulty understanding (24). Participants received gift cards or cash for each check-in ($5) and interview ($20).

We identified HOPE HOME study participants who remained active in the study between November of 2020 and February of 2021 and who reported that they first became homeless at age 50 and older and had an active phone number (*n* = 100). We offered recruitment to the first 35 individuals on a list of participants ordered sequentially by latest follow-up date. We were able to enroll 27 consecutive participants, 25 of whom completed all components of this study: structured neurocognitive history intake, neurological examination, and neuropsychological examination. Two participants were unable to complete all components due to inability to travel to site for in-person evaluation.

We conducted interviews and exams between November 2020 and February 2021. We offered a $30 debit card for neurocognitive history and an additional $30 for the neurological and neuropsychological examinations.

Due to research restrictions imposed by the Coronavirus Disease 2019 (COVID-19) pandemic, we conducted in-person evaluations outdoors in a private setting outside the HOPE-HOME study site in Oakland, California. Whenever possible, we conducted neurocognitive history intakes over the telephone.

### Data storage

We collected de-identified data (assigning each individual a personalized ID number) in paper forms. These forms were stored securely and discarded after being scanned into a secure electronic database.

### Study measures

#### Functional status

We used data on functional status from the most recent HOPE HOME semi-annual interview that was conducted prior to each participant's neurocognitive evaluation for this study. As part of HOPE HOME study procedures, participants reported whether they received support from anyone on a daily basis to help with instrumental and/or non-instrumental activities of daily living (iADL and ADLs). Participants reported whether they had difficulty with everyday activities because of a physical, mental, emotional, or memory problem: dressing, bathing, or showering, eating, getting in or out of bed, using the toilet, and walking across the room (25). If a participant could not perform an activity because of lack of access to resources, the interviewer determined the participant could otherwise perform said activity. HOPE HOME also used the Brief Instrumental Functioning Scale (BIFS) to determine iADLs as it has been validated in homeless populations (25–27). Staff asked participants if in the past 4 weeks they could perform each activity on their own, with help, with a little help, with a lot of help, or needing someone to do the tasks for them. These activities included: (1) Taking medications as prescribed by a doctor, (2) filling out an application for benefits, (3) keeping up with or budgeting money, (4) using public transportation, (5) setting up a job interview by phone, and (6) finding an attorney to help with a legal problem. We scored BIFS as binary. The mean duration between time of most recent functional assessment and time of neurocognitive evaluation for this study was 3 months prior to our neurological examination.

#### Alcohol use, drug use, and depression measures

HOPE HOME staff collected data on alcohol use, drug use, and depression using validated scales in their 6-month follow-up visits. We used the closest score to our neurologic assessment and excluded individuals with assessments more than 4 weeks after our neurologic exam.

The HOPE HOME staff used the Alcohol Use Disorders Identification Test (AUDIT) (28) to assess for alcohol use disorders. HOPE HOME modified the AUDIT by asking about behaviors in the past 6 months, instead of past year. We considered scores of 8–15 as measures of harmful alcohol use, 16–19 as moderate disorders, and 20 and above as evidence of severe alcohol use disorders.

HOPE HOME staff also adapted questions from World Health Organization Alcohol, Smoking, and Substance Involvement Screening Test (ASSIST) (29, 30) which assesses drug use in the 12 months prior to survey administration to assess drug use in the past 6 months. We asked participants how often they (1) used, (2) had a strong desire or urge to use, (3) experienced health, social, legal or financial problems as a result of using, and (4) failed to do what was normally expected of them due to using the following substances: cannabis, cocaine, amphetamine type stimulants, inhalants, sedatives or sleeping pills, hallucinogens, opioids, or other drugs in the past 6 months. Possible responses (and assigned scores) were the following: never (0), once or twice (2), monthly (3), weekly (4), and daily or almost daily (6). HOPE HOME asked participants if a friend, relative, or anyone else had ever expressed concern about their drug use for the listed substances and if they ever tried and failed to control, cut down, or stop using each listed drug. Possible responses (and assigned score) were never (0), in the past 6 months (6), and not in the past 6 months (3). For each substance, we specified substance involvement risk as lower risk (score 0–3), moderate risk (score 4-26), and high risk (score 27+).

HOPE HOME used the Center for Epidemiologic Studies Depression Scale (CES-D) (31) to assess depression in participants, which has shown to be a reliable measure of depression in homeless populations. We asked participants if they experienced various feelings or behaviors rarely (not at all or <1 day, score of 0), some or a little of the time (1–2 days, score of 1), occasionally or a moderate amount of time (3–4 days, score of 2), or most or all of the time (5–7 days, score of 3). We asked participants about the following experiences: (1) being bothered by things they usually aren't bothered by, (2) poor appetite, (3) not being able to shake off the blues even with help from family/friends, (4) feeling just as good as other people, (5) having trouble keeping his/her mind on task, (6) feeling depressed, (7) feeling like everything is an effort, (8) feeling hopeful about the future, (9) thinking his/her life had been a failure, (10) fear, (11) restless sleep, (12) happiness, (13) talking less than usual, (14) loneliness, (15) feeling that people were unfriendly, (16) enjoying life, (17) crying spells, (18) sadness, (19) feeling that people disliked him/her, (20) feeling that he or she could not get “going.” Scores of 0–3 for each of the 20 questions were combined for a total score of up to 60. We used a standard threshold score of 16 or more to categorize possible clinical depression.

#### Neurocognitive history

Neurologists [SM and SL] conducted structured neurocognitive histories in all study participants through a phone interview. This included participants' age, sex, self-reported race, educational attainment, and work experience/stated profession. We also screened participants for comorbidities that are known risk factors for NDDB and/or cognitive decline: traumatic brain injury (TBI), stroke/transient ischemic attack, seizures/epilepsy, encephalitis/meningitis, sleep disorders (sleep apnea, rapid eye movement sleep behavior disorder), hypertension, hypercholesterolemia, obesity, diabetes, hearing loss, vision loss, cardiovascular disease, and thyroid disorders (32). Along with formalized scores for measuring alcohol use and drug use (methamphetamine, opioid, cocaine, and cannabis), we obtained informal data on individuals' use, we defined remote alcohol and illicit drug use as no use within the previous year (per self-report). We screened for the presence of known specific neurodegenerative disorders among first- and second-degree relatives (Alzheimer disease, Parkinson disease, Lewy body dementia, frontotemporal dementia, amyotrophic lateral sclerosis/Lou Gehrig's disease, Pick's disease, vascular dementia, and any other forms of dementia and neurological disease). We asked the following question to elicit any other pertinent family history from each participant: “Did anyone in your family, including grandparents, parents, aunts or uncles, cousins, brothers or sisters, or children experience progressive loss of mental functions, or thinking abilities, or cognitive functions? Progressive changes in personality or behavior? Progressive difficulties speaking? Progressive difficulties using their arms and legs?”

We [SM and/or SL] then assessed each participant's neurocognitive history following a structured interview based on gold standard evaluation procedures in the UCSF ADRC. Participants were asked to report on perceived changes to their neurocognitive health over the previous “few years” compared to their perceived baseline. The interview began with a brief assessment of subjective cognitive decline (SCD) based on a previously published SCD interview, the SCD-I (33). This interview begins with an open-ended question (“During the past few years, have you noticed any changes in your mental abilities? Could you give an example from everyday life”), followed by a brief structured assessment of changes to individual cognitive domains and an assessment of when said cognitive changes (if any) began, whether or not the participant felt concerned about said cognitive changes (relative to self-perceived baseline and perceived cognitive abilities of age-matched peers), and if the participant sought medical care for said complaints. This initial assessment was followed by a detailed neurocognitive review of systems that probed the following domains: episodic memory (remote and recent), visuospatial skills (orientation/navigation, judgment of depth/distance, problems with visual perception), executive function (planning/organization, multi-tasking, attention span), language (expression and comprehension), sleep, autonomic, and sensory functions, motor function (gait, falls, weakness, involuntary movements), and behavioral and emotional-processing changes.

#### Neurological examination

Participants were scheduled for in-person neurological examinations after completing their phone-based neurocognitive history examinations. Experienced neurologists [SM or SL] performed neurologic examinations in a safe and private outdoor space within the HOPE-HOME study site in Oakland, California. Neurological examinations were conducted using Personal Protective Equipment (PPE) in accordance with COVID-19 public health recommendations. In addition to all components of a gold standard bedside neurological examination (cranial nerves, motor systems, coordination, sensory systems, deep tendon reflexes, and gait examination) we assessed olfaction using the Brief Smell Identification Test (BSIT) (34) given that hyposmia/anosmia is a known risk factor for NDDB, especially alpha-synuclein associated disease (35).

#### Neuropsychological examination

Trained testers [SM or GA] conducted all neuropsychologic assessments, which were administered on the UCSF Tablet-based Cognitive Assessment Tool (TabCAT) platform (36, 37). The tests were developed and validated by UCSF neuropsychologists to measure cognitive skills that are affected by typical and atypical presentations of NDDB (37, 38). Participants were asked to report any drug use prior to test administration and were assessed for clinical signs of intoxication. If participants screened positive, they were re-scheduled for a different visit. None of the participants in this study showed clinical signs of intoxication during testing. Memory was assessed using Favorites, an associative memory test that requires participants to learn associations between verbal and visual stimuli. Performance was summarized by the total correct across the 2 learning and 1 delay trials. Executive functions were assessed by Match, which requires participants to quickly match numbers (1–7) and simple pictures. Performance was summarized by total correct in 2 min. Flanker (inhibitory attention) and Dot Counting (verbal working memory) tests from the NIH EXAMINER battery provided additional executive assessments (39, 40). Visuospatial skills was measured by Line Orientation, which requires participants to indicate which of two lines is parallel to a target line. Language was assessed by Animal Fluency (41). Social cognition was assessed with the Dynamic Affect Recognition Test (DART) on which participants are asked to identify the emotion expressed in each of a series of short videos relying on nonverbal cues. In total, administration of the TabCAT spanned 30–45 min (42).

### Data analysis

Participants' scores on neuropsychological testing were adjusted for demographic factors using normative data (42). For Favorites, Match, Line Orientation, and Animal Fluency, the scores were corrected for age and education level using a previously published regression approach (38). Flanker and Dot Counting were corrected for age and sex using the same method. DART was corrected for sex and age using a traditional box norms approach. As the participant sample is disadvantaged relative to reference samples, a conservative impairment threshold (z-scores < −2.0) was selected.

Using the summation of data obtained from structured neurocognitive history, neurological examination, neuropsychological examination, and functional assessment, we assigned each participant into one of four groups to denote each participant's overall neurocognitive health status: neurocognitively normal, SCD (43), and mild or major neurocognitive disorder based on criteria from the 5th version of the Diagnostic and Statistical Manual (44, 45). “Neurocognitively normal” participants were those that did not report or endorse neurocognitive concerns based on our neurocognitive history intake, performed within normal range on neuropsychological examination, and reported intact ADLs and iADLs. Participants with SCD were those who reported and/or endorsed neurocognitive concerns on our neurocognitive history intake but performed within normal range on neuropsychological examination and reported intact ADLs and iADLs. Participants with mild neurocognitive disorder (MiNCD) were those who reported and/or endorsed neurocognitive concerns on our neurocognitive history intake and performed below expected on neuropsychological examination yet reported intact ADLs and iADLs. Finally, participants with major neurocognitive disorder (MaNCD) were those who reported and/or endorsed neurocognitive concerns on our neurocognitive history intake, performed below expected on neuropsychological examination, and reported significant impairments on their ADLs and/or iADLs.

Subsequently, we screened all neurocognitive signs and symptoms obtained from structured neurocognitive history, neurological examination, neuropsychological examination, and functional assessment, to assign each participant into one or more of the gold standard research diagnostic criteria for NDDB, which included the following criteria: possible Alzheimer's Dementia (AD), possible behavioral variant frontotemporal dementia (BvFTD), probable corticobasal syndrome (CBS), possible CBS, possible Lewy body dementia (DLB), amnestic mild cognitive impairment (MCI), non-amnestic MCI, possible multiple system atrophy (MSA), primary progressive aphasia (logopenic variant, semantic variant, and/or agrammatic variant), possible Parkinson's disease (PD), possible progressive supranuclear palsy (PSP), suggestive PSP, and posterior cortical atrophy (PCA) (46–56). In this manner, we explored possible etiologic diagnoses for the observed neurocognitive deficits observed in participants based solely on clinical data.

## Results

### Pertinent sociodemographic characteristics, medical, and family history

Participants ranged in age from 54 and 71 years, with a median age of 61. The majority of participants were male (76%), reported high school/GED level of education and above (84%), and were Black (84%). Most participants (*n* = 19, 76%) were currently housed at the time of interview. With regards to pertinent family history, nearly half of participants (44%) reported a family history of suspected NDDB, with AD being the predominant suspected etiology. Participant medical history was notable for a higher prevalence of cardiovascular disease (hypertension, heart disease, hyperlipidemia, diabetes) than the general population (47% of hypertension, 10% diabetes, and 12% hyperlipidemia in the general population, per Centers for Disease Control and Prevention, CDC) (57–59). Moreover, 40% of participants reported a history of depression (vs. 8.4% in the general population, per CDC) with at least 17% noting current moderate to severe current symptoms per the CES-D (60). Close to 25% of participants reported a history of TBI (vs. 0.3% in the general population, per CDC) (61). Over half of participants (56%) reported a history of illicit substance use (though only 20% of individuals endorse current use with cocaine and cannabis being the commonly used substance per ASSIST scores), 60% reported a history of tobacco use, and 40% reported a history of alcohol use. Rates of alcohol use and illicit drug use are lower in the general population (6% heavy alcohol use and 21.4% illicit drug use) (60) (Table 1).

**Table 1:** Pertinent sociodemographic characteristics, medical and family history (based on self-report).

**Sociodemographic and health characteristics**	***N***, **%**
**Sex**	
Female	6, 24
Male	19, 76
**Race/ethnicity**	
Black	21, 84
Other	4, 16
Non-hispanic white	1, 4
Hispanic	1, 4
Mixed race	2, 8
**Education**	
Less than high school/GED	4, 16
Finished high school	15, 60
Completed junior college/college	5, 20
Completed graduate school	1, 4
Current housing status	
Housed	19, 76
Unhoused	6, 24
**Family history of neurologic disease**	
Alzheimer's disease/Unspecified dementia	11, 44
Frontotemporal dementia	0, 0
Parkinson's disease	0, 0
Dementia with Lewy bodies	0, 0
Pick's disease	0, 0
Vascular dementia	0, 0
Amyotrophic lateral sclerosis/Lou Gehrig's disease	1, 4
**Drug use**	
History of methamphetamine, cocaine, marijuana, and/or opioid use	14, 56
WHO-ASSIST, Opioids^*^ moderate risk (4-26)	1, 4
WHO-ASSIST- Methamphetamine^*^ Moderate Risk (4-26)	4, 17
WHO-ASSIST- Cocaine^*^ Moderate Risk (4-26)	21, 88
WHO-ASSIST- Cannabis^*^ Moderate Risk (4-26)	21, 88
Remote heavy alcohol use (>3 drinks/day), currently sober	4, 16
AUDIT Score^*^: Medium-High (8-20)	4, 17
**Medical comorbidities that are pertinent to brain health**	
Hypertension	16, 64
Heart disease	5, 20
Hyperlipidemia	9, 36
Diabetes	5, 20
Stroke/transient ischemic attack (TIA)	1, 4
Seizures/Epilepsy	0, 0
Thyroid disorder	0, 0
Encephalitis/Meningitis	0, 0
Rapid eye movement sleep behavior disorder	0, 0
Sleep apnea	2, 8
History of depression	10, 40
Moderate/severe CES-D Score (22+)^*^	4, 17
**Traumatic brain injury**	
With loss of consciousness (LOC)	2, 8
Without LOC	4, 16
Current tobacco use	15, 60

* *2 individuals did not have scores for AUDIT, ASSIST, and CES-D within an appropriate time window and thus their scores are not reported*.

### Neurocognitive history

Approximately half of participants (*n* = 13, 52%) reported neurocognitive deficits based on the SCD-I; the majority of these (*n* = 9, 69%) expressed feeling worried about their perceived cognitive deficits, yet none of the participants had seen a doctor or health provider to address their concerns. The average age of first symptom onset was 56 years with an average of 5.1 years (SD = 3.12) since first symptom on interview. Those who endorsed symptoms mostly noted deficits in memory, executive function, and language.

All participants endorsed neurocognitive changes compared to their perceived baseline across various neurocognitive domains assessed, with changes in the behavioral/socioemotional domain being most frequently endorsed. We found that 80% of participants (*n* = 20) noted changes in motivation and/or apathy, while only 32% (*n* = 8) perceived changes to their overall emotional health. Within the domain of language function, more than half of participants (*n* = 16, 64%) endorsed deficits in language expression (word finding difficulties being most common). There was a high prevalence of recent episodic memory loss (*n* = 15, 60%) and executive dysfunction with difficulties in planning and organization being most prevalent (*n* = 13, 52%). Although visuospatial deficits were less commonly reported, 16% of participants endorsed experiencing difficulties with navigation/orientation (*n* = 4), and only two participants endorsed hallucinations and/or visual perception changes (Table 2).

**Table 2:** Neurocognitive signs and symptoms based on structured neurocognitive review of systems.

**Neurocognitive domain**	***N***
**Episodic memory functions**	
Recent	15, 60
Remote	1, 4
**Visuospatial processing functions**	
Difficulties with navigation/orientation	4, 16
Difficulty judging depth/distance	3, 12
Difficulty with visual perception (including illusions and hallucinations)	2, 8
**Executive functions**	
Difficulty with planning/organization	13, 52
Difficulty multi-tasking/parallel tasking	8, 32
Poor concentration/attention span	11, 44
**Language functions**	
Impaired language expression	16, 64
Impaired language comprehension	3, 12
**Sleep health**	
Insomnia	12, 48
Sleeping more/napping more	11, 44
Fluctuating wakefulness/attention during day	4, 16
Loud snoring	10, 40
Apneic episodes	3, 12
Acting out dreams	7, 28
**Motor functions**	
Gait difficulties	8, 32
Falls	7, 28
Difficulty using hands	3, 8
Muscle weakness	6, 24
Involuntary movements	3, 12
Twitches	1, 4
Jerks	0, 0
Tremors	2, 8
Dysphagia	1, 4
Autonomic/sensory changes	16, 64
Lightheadedness/dizziness	4, 16
Erectile dysfunction	2, 11
Constipation	3, 12
Urinary incontinence	2, 8
Anosmia	2, 8
Hyper/hypo hidrosis	3, 12
Neuropathy	10, 40
**Behavioral/socioemotional functions**	
Changes in emotional health	8, 32
Changes in behavior	14, 52
Disinhibition	4, 16
Binge eating	7, 28
Change in food preferences	2, 8
Lack motivation/apathy	20, 80

Sensory and autonomic changes, including constipation, erectile dysfunction, urinary changes, numbness, and tingling, were common among 16 participants (64%); most common amongst these were sensory changes, with 10 participants (40%) reporting numbness/tingling in either hands or legs. Eight individuals (32%) endorsed gait difficulties; 7 participants (28%) endorsed experiencing falls. Involuntary movements were uncommon. Two individuals noted tremor while only one individual noted twitching. While sleep health concerns were less common than cognitive concerns, seven participants were aware of possible dream enactment behaviors (28%), 12 participants reported experiencing insomnia (48%), and 11 participants reported increased daytime sleepiness (44%).

### Neurological examination

Among the 19 participants who took the BSIT, four (21%) had abnormalities in the sense of smell. Of note, four participants (16%) exhibited mild impairments in extraocular movements, three participants exhibited pupillary function abnormalities, and one participant presented with a cortical visual field cut. No other cranial nerve abnormalities were detected (Table 3).

**Table 3:** Neurological signs based on neurological examination.

Neurological signs	***N***, **%**
**Cranial nerve abnormalities**	
Hyposmia/anosmia^*^	4, 21
Visual field cut	1, 4
Pupillary abnormalities	3, 12
Extraocular movement abnormalities	4, 16
Diminished facial sensation	1, 4
Hearing loss	1, 4
**Motor function abnormalities**	
Weakness	1, 4
Increased tone	6, 24
Tremor	2, 8
Fasciculations	1, 4
**Coordination abnormalities**	
Upper extremity clumsiness/Slow finger taps	11, 44
Lower extremity clumsiness/Slow foot taps	4, 16
**Breakdown of set**	
Pronation/Supination	14, 56
Opening/Closing Fist	13, 52
Luria sequence abnormalities	16, 64
Dysmetria on heel-knee-shin	1, 4
Apraxia of upper and/or lower extremities	2, 8
**Sensory function abnormalities**	
Cortical	1, 4
Peripheral	8, 32
**Deep tendon reflex abnormalities**	
Increased	3, 12
Decreased	16, 64
**Gait abnormalities**	
Parkinsonism	5, 20
Retropulsion (Positive Pull Test)	2, 8
Difficulties with tandem gait	8, 32
Wide-based gait	5, 20

* *BSIT performed on 19 participants (76%)*.

The most common finding observed on motor exam was increased tone (*n* = 6, 24%). This presented as unilateral, upper extremity cogwheeling in five of these individuals, and increased tone in the bilateral legs in one individual. One individual had fasciculations in his back and calves that have been present since spinal surgery, and two participants had a non-resting tremor of the bilateral upper extremities. Difficulties performing the Luria task were common (*n* = 16, 64%), as well as a breakdown of set on pronation/supination of hands (*n* = 14, 56%) and opening/closing of fists (*n* = 13, 52%). The majority of participants exhibited clumsy and/or slowed finger taps (*n* = 11, 55%). Signs of apraxia were uncommon, with only two participants demonstrating signs of appendicular upper extremity apraxia (8%).

On sensory examinations, nearly one-third of participants (*n* = 8, 32%) had bilateral length-dependent and ascending polyneuropathy at the feet to the shins, and over half of participants (*n* = 16, 64%) demonstrated diminished ankle jerk reflexes, again suggestive of an underlying neuropathy. Conversely, three participants (12%) exhibited hyperreflexia at the patella and/or asymmetric reflexes in the upper extremities.

On gait examination, findings were varied but not strongly prevalent. Approximately one third of participants (*n* = 8, 32%) had difficulties with tandem gait, while five participants (20%) had either a parkinsonian gait or wide-based gait. Testing of retropulsion was positive in two participants (8%).

### Neuropsychological examination

All participants exhibited signs of cognitive impairment based on TabCAT. In agreement with self-report of neurocognitive deficits obtained through detailed neurocognitive history, a majority of participants (*n* = 17, 68%) exhibited socioemotional deficits on testing. Executive dysfunction and memory deficits were also common, with 16 participants (64%) exhibiting signs of executive dysfunction and 12 participants (48%) exhibiting signs of impaired recent episodic memory function. Of note, performance outcomes were unavailable for 6 participants on the Flanker, an executive function task, due to difficulty completing practice trials. Almost one-third had visuospatial deficits (*n* = 8, 32%), and only two participants had deficits on verbal fluency (8%) (Table 4).

**Table 4:** Neuropsychological deficits based on TabCAT.

**Neurocognitive domain**	***N***, **%**
**Episodic memory impairments**	
Favorites	12, 48
**Executive function impairments**	
Match	16, 64
Dot counting	8, 32
Flanker	5, 20
**Language deficits**	
Animal fluency	2, 8
**Visuospatial deficits**	
Line orientation	8, 32
**Socioemotional deficits**	
DART	17, 68

### Functional independence

At least a quarter (*n* = 7, 28%) of participants in this study reported any ADL deficit while close to a third (*n* = 8, 32%) reported any iADL deficit on structured history. Twelve percent (*n* = 3) of participants endorsed functional deficits on the BIFS.

### Degree of neurocognitive impairment and possible etiologies

None of the participants in this study were considered to be neurocognitively normal; and because all participants exhibited deficits on neuropsychological testing, none met criteria for SCD. Four participants could not be classified because they did not volunteer or endorse neurocognitive concerns or complaints and denied functional impairments despite exhibiting signs of neurocognitive impairment on examination.

Ten participants (40%) reported/endorsed neurocognitive deficits that were substantiated on neuropsychological testing and were severe enough to interfere with activities of daily living, thus meeting criteria for MaNCD. A similar proportion of participants (*n* = 11, 44%) reported/endorsed neurocognitive deficits that were substantiated on neuropsychological examination but were not severe enough to interfere with activities of daily living, thus meeting criteria for MiNCD. These participants did not meet any exclusion criteria for MiNCD or MaNCD.

We only included diagnostic categories that did not require biomarker data (including neuroimaging). Eight participants had neurocognitive deficits but no clear pattern of decline (i.e., they met criteria for many subtypes of NDDB) or had co-morbidities (i.e., stroke) that clouded their assessment. MCI was most common, with five participants meeting criteria for the amnestic variant and two individuals meeting criteria for the non-amnestic variant. The incidence of bvFTD and AD was identical (*n* = 4, 16%). There was a low incidence of possible DLB (*n* = 2, 8%). None of the participants met criteria for CBS, MSA, PCA, PD, PPA, or PSP (Table 5).

**Table 5:** Possible etiological diagnosis, based on clinical research criteria.

**Clinical research diagnosis**	***N, %***
Possible bvFTD	4, 16
Possible AD	4, 16
Possible DLB	2, 8
MCI	7, 28
*Amnestic*	5, 20
*Non-Amnestic*	2, 8
Probable CBS	0, 0
Possible CBS	0, 0
Possible MSA	0, 0
PPA	0, 0
Possible PD	0, 0
Possible PSP	0, 0
Suggestive PSP	0, 0
Indeterminant	8, 32

## Discussion

In this neurocognitive health study of older homeless adults with a first episode of homeless at age 50 and older, we found that self-reported neurocognitive concerns were highly prevalent and well-substantiated on structured neurological and neuropsychological examinations. None of the participants in this study were deemed neurocognitively normal, and nearly half met criteria for mild or major neurocognitive disorder based on detailed clinical examinations. More than half of participants in this study met clinical research criteria for specific neurodegenerative disorders.

We found that several general health conditions that are relevant to brain health were highly represented among our study participants, especially hypertension, diabetes, hyperlipidemia, depression, smoking, and traumatic brain injury. All these conditions have been specifically associated with risk of NDDB (9), and previous studies have shown that they are highly prevalent in older adults experiencing homelessness (27). In studies specific to FTD, TBI, obesity, diabetes, hypertension, and hyperlipidemia have all been associated with FTD risk to varying degrees (62–64). Further longitudinal studies are needed to ascertain the cumulative impact of these and other general health conditions on specific brain health outcomes among unhoused older adults, including risk of NDDB and cerebrovascular disease.

We elicited a wide range of focal neurocognitive complaints/concerns in our study participants, including behavioral and emotional changes, with signs and symptoms of frontotemporal neurocognitive impairment most common. We found a high prevalence of self-reported depression (40%), higher than housed adults 65 years and older (5–10%) (65), as well as perceived changes in emotional health (32%), changes in behavior (52%), and lack of motivation/apathy (80%). These are noteworthy observations, as it is well-known that late-life mood symptoms (anxiety and depression), as well as behavioral changes (apathy and disinhibition) may represent early manifestations of an underlying NDDB, particularly DLB, AD, and FTLD. Moreover, such changes in mood and behavior often manifest years before signs or symptoms of cognitive decline (i.e., memory loss, executive dysfunction, etc.) emerge. Longitudinal studies on AD, for example, show that apathy, irritability, and depression are common prodromal features of this disease (66). And persons with bvFTD frequently exhibit apathy and anhedonia, or disinhibition early in their illness—signs and symptoms that are often misinterpreted as major depression or other psychiatric illness early in the disease course (6, 67). Mid to late life mood disorders in unhoused older adults, especially in the setting of absent early life psychiatric illness, should raise concern for a an early NDDB.

Many participants in our study who did not volunteer neurocognitive signs and symptoms on open-ended questioning did endorse neurocognitive deficits on structured history intake, suggesting that a detailed and structured neurocognitive history intake approach is be needed to elicit these clinically significant deficits in this population.

The predominance of frontotemporal signs and symptoms captured in our study participants based on neurocognitive history was corroborated on neuropsychological testing. We found a high prevalence of socioemotional processing deficits, as well as signs of executive dysfunction and associative memory dysfunction. These deficits provide evidence for frontotemporal lobar dysfunction, in agreement with findings from our retrospective study on homelessness among persons with NDDB (8). Moreover, our study results also suggest a relative sparing of parieto-occipital lobe functions, based on both history and neuropsychological testing. Characterizing the patterns of neurocognitive impairment is important for diagnostic and prognostic reasons, as persons with typical AD, for example, demonstrate deficits that localize to the temporo-parietal regions of the brain, whereas persons with FTLD and atypical AD variants typically demonstrate deficits that localize to anterior frontotemporal brain regions (7, 68, 69).

We observed a wide range of focal neurological deficits in our study participants based on detailed neurological examinations. Most notable were deficits in cranial nerve function, motor function, coordination, and peripheral sensory functions, thus indicating a mix of central and peripheral neurological deficits. Such multifocal localization suggests the possibility of diverse neurological injuries among older adults experiencing homelessness. The high prevalence of history of illicit drug use and alcohol use among participants in this study may partly explain these findings. Heavy alcohol and illicit substance use have well-studied neurologic effects. Cocaine use disorder, for example, can disrupt nigrostriatal dopamine transmission leading to impairments in motor function and coordination such as those observed in this study, and can also impair the integrity of white matter tracts through vasoconstriction of cerebral vessels and hypoperfusion of brain parenchyma (70). Moreover, illicit drug abuse can also lead to structural brain changes. On post-mortem autopsies of regular methamphetamine users, for example, significant hippocampal volume loss has been observed (70).

Alternatively, focal neurological examination deficits are also commonly observed in persons with early NDDB, hence the neurological deficits we observed in our study participants raise concern for unrecognized primary neurological disease in unhoused older adults. For example, reduced sense of smell in the setting of signs and symptoms concerning for autonomic nervous system dysfunction (orthostatic hypotension, constipation, erectile dysfunction, etc.) and rapid eye movement (REM) sleep behavior disorder—all of which were observed in our study participants—raises concerns early underlying Parkinson disease (PD) or dementia with Lewy body (DLB) disease processes (71). In this regard, it is also noteworthy that several of our study participants presented with signs of parkinsonism on neurological examination (bradykinesia, limb rigidity, gait instability, tremor, etc.), which would also raise concern for PD or DLB (72, 73). Moreover, atypical presentations of AD, which are most common in persons younger than 65, can also present with signs of Parkinsonism years before the onset of cognitive impairment (74). Neurological longitudinal research studies are needed to determine the clinical significance of these neurological signs and symptoms in homeless older adults.

None of the participants in this study were neurocognitively normal, and none met criteria for SCD because all participants exhibited deficits on neuropsychological testing and/or reported deficits in ADLs and/or iADLs. Most participants in our study (84%) met criteria for MiNCD or MaNCD. These disorders do not necessarily lie on a continuum. Independent reversible or irreversible etiologies of MiNCD and MaNCD exist and not all persons with MiNCD progress to MaNCD. Still, both disorders portend an unfavorable prognosis if not clinically evaluated and addressed. This is particularly relevant to the experience of homelessness, as a homeless older adult with MiNCD or MaNCD due to an underlying NDDB will eventually need individualized, lifelong support to find and maintain stable housing and avoid institutionalization as their disease progresses. This is because all forms of NDDB progress insidiously and irreversibly, thus leading affected individuals to become dependent on others for ADLs and iADLs, as well as for medical, legal, and financial decisions and procedures.

While all participants in our study exhibited impairments on neuropsychological testing, four did not report or endorse any neurocognitive complaints or functional decline on history intake. Therefore, they did not meet criteria for SCD, MiNCD, or MaNCD; they were also not considered to be neurocognitively healthy, however. Instead, we suspect these four participants did not report or endorse neurocognitive or functional decline on history intake due to lack of insight into their neurocognitive health. Lack of insight, also known as anosognosia, is common in NDDB, particularly FTLD subtypes. Lack of insight is also characterized as a lack of concern, or anosodiaphoria, in the neuropsychiatric literature. In a study of 29 individuals with known FTLD, most individuals denied having a problem that required medical attention while simultaneously being able to narrate the behaviors they have that society would consider problematic. This phenomenology is commonly observed among persons with FTLD, and it localizes to the right frontal lobe (75).

Guided by current research criteria for neurocognitive disorders, we found that approximately 16% of our study participants met criteria for possible bvFTD and 16% for possible AD, whereas 8% met criteria for possible DLB. Approximately 28% of our study participants met criteria for MCI, about one third of these were non-amnestic. Although lack of longitudinal data, including reliable informants, and lack of brain health biomarkers greatly limited our ability to characterize these individuals more precisely, we found that the signs and symptoms elicited from our participants based on detailed history and neuropsychological testing conformed mostly to AD and FTLD syndromes, whereas other neurodegenerative syndromes such as PD, progressive supranuclear palsy, corticobasal syndrome, and primary progressive aphasia, were less common in our study. These findings support our prior study results (8) and our overarching hypothesis that frontotemporal neurocognitive syndromes are prevalent in older adults experiencing homelessness.

### Strengths, limitations, and future directions

The main strength of the present study pertains to its approach to studying neurocognitive health in older adults experiencing homelessness. We are not aware of any other similar studies that have employed a domain-specific behavioral neurology approach to the study of brain health in persons experiencing homelessness. In future studies, we aim to expand and deepen this approach by including brain health biomarkers and longitudinal neurological and more comprehensive neuropsychological assessments.

In terms of weaknesses and limitations, our study population is small, and most participants were Black, male, and fluent in English, hence our study population and results are not representative of the larger population of unhoused older adults. Given the high prevalence of substance use among the homeless population, we decided not exclude participants based on self-reported history of substance use and instead used clinical judgment to exclude participants who were showing obvious signs of intoxication. It is thus possible substance use may account for observed neurocognitive findings in some participants. Furthermore, it is possible that undetected/undiagnosed COVID-19 influenced some of the neurocognitive manifestations observed in some participants. Due to restrictions imposed by COVID-19, we were only able to recruit and evaluate participants who were able to use a phone, hence our results may underestimate the degree of neurocognitive impairment in our study population. Furthermore, those with problematic drug and/or alcohol use were likely under-sampled because they either had died prior to this sub-study's recruitment or were unable to be consented for and/or organize themselves to participate in this sub-study.

The present pilot study lays the foundation for the development of a longitudinal study of neurocognitive health among unhoused older adults in San Francisco in collaboration with community-based centers that serve this population. Such a study is necessary because most forms of NDDB have years long natural histories, hence longitudinal neurological studies are needed to investigate brain health as both precipitant to, and recipient of, the experience of homelessness among older adults. In addition to detailed clinical and neuropsychological assessments that are gold standard within the field of behavioral neurology and neuropsychiatry, we intend to add brain health biomarkers, including imaging and serum markers of neurodegeneration, to characterize the overall brain health of this population more deeply. We hope that through this longitudinal approach we will be able to contribute to our understanding of the causes and consequences of homelessness and inform person-centered support and policies to prevent and address this complex condition.

## Data availability statement

The raw data supporting the conclusions of this article will be made available by the authors, without undue reservation.

## Ethics statement

The studies involving human participants were reviewed and approved by University of California, San Francisco IRB. The patients/participants provided their written informed consent to participate in this study.

## Author contributions

SM, SL, and MK contributed equally to the formulation of the original idea, data collection, data analysis, and composition of the final manuscript. SC, JW, SK, GA, LL, ET, KV, KP, CG, and BM contributed equally to formulation of original data, data collection, and analysis. All authors contributed to the article and approved the submitted version.

## Funding

This work was supported by R01AG041860 and K24AG046372 from the National Institute on Aging at the National Institutes of Health (MK), the UCSF Benioff Homelessness and Housing Initiative (MK), the UCSF ADRC (SL), the Population Health Equity Scholar Award (SL), and the Roland Nyegaard Endowed Professorship in Vulnerable Populations (SL). The use of the TabCAT (KP, ET, and CG) was supported by UG3 NS105557-01 (KP) from the National Institute of Neurological Disorders and Stroke at the National Institutes of Health.

## Conflict of interest

The authors declare that the research was conducted in the absence of any commercial or financial relationships that could be construed as a potential conflict of interest.

## Publisher's note

All claims expressed in this article are solely those of the authors and do not necessarily represent those of their affiliated organizations, or those of the publisher, the editors and the reviewers. Any product that may be evaluated in this article, or claim that may be made by its manufacturer, is not guaranteed or endorsed by the publisher.

## References

[1] Sermon MMH. Demographics of Homelessness Series: The Rising Elderly Population. Washington, DC: Homelessness Research Institute (2010).

[2] Culhane D Treglia D Byrne T Metraux S Kuhn R Doran K. Emerging Crisis of Aged Homelessness. Actionable Intelligence for Social Policy (AISP) (2019).34746445

[3] Montgomery AE Cutuli JJ Evans-Chase M Treglia D Culhane DP. Relationship among adverse childhood experiences, history of active military service, and adult outcomes: homelessness, mental health, and physical health. Am J Public Health. (2013) 103(Suppl 2):S262–8. 10.2105/AJPH.2013.301474 24148064 PMC3969137

[4] Plassman BL Langa KM Fisher GG Heeringa SG Weir DR Ofstedal MB. Prevalence of dementia in the United States: the aging, demographics, and memory study. Neuroepidemiology. (2007) 29:125–32. 10.1159/000109998 17975326 PMC2705925

[5] Liljegren M Naasan G Temlett J Perry DC Rankin KP Merrilees J. Criminal behavior in frontotemporal dementia and Alzheimer disease. JAMA Neurol. (2015) 72:295–300. 10.1001/jamaneurol.2014.3781 25559744 PMC4432918

[6] Lanata SC Miller BL. The behavioural variant frontotemporal dementia (bvFTD) syndrome in psychiatry. J Neurol Neurosurg Psychiatry. (2016) 87:501–11. 10.1136/jnnp-2015-310697 26216940 PMC4755931

[7] Seeley WW Crawford RK Zhou J Miller BL Greicius MD. Neurodegenerative diseases target large-scale human brain networks. Neuron. (2009) 62:42–52. 10.1016/j.neuron.2009.03.024 19376066 PMC2691647

[8] Piña-Escudero SD López L Sriram S Longoria Ibarrola EM Miller B Lanata S. Neurodegenerative Disease and the Experience of Homelessness. Front Neurol. (2020) 11:562218. 10.3389/fneur.2020.562218 33519660 PMC7838483

[9] Livingston G Huntley J Sommerlad A Ames D Ballard C Banerjee S. Dementia prevention, intervention, and care: 2020 report of the Lancet Commission. Lancet. (2020) 396:413–46. 10.1016/S0140-6736(20)30367-6 32738937 PMC7392084

[10] Gelberg L Linn LS Usatine RP Smith MH. Health, homelessness, and poverty. A study of clinic users. Arch Intern Med. (1990) 150:2325–30. 10.1001/archinte.1990.00390220069014 2241441

[11] Gelberg L Linn LS. Demographic differences in health status of homeless adults. J Gen Intern Med. (1992) 7:601–8. 10.1007/BF02599198 1453243

[12] Solliday-McRoy C Campbell TC Melchert TP Young TJ Cisler RA. Neuropsychological functioning of homeless men. J Nerv Ment Dis. (2004) 192:471–8. 10.1097/01.nmd.0000131962.30547.26 15232317

[13] Stone B Dowling S Cameron A. Cognitive impairment and homelessness: a scoping review. Health Soc Care Community. (2019) 27:e125–e42. 10.1111/hsc.12682 30421478 PMC6849546

[14] Gicas KM Jones AA Thornton AE Petersson A Livingston E Waclawik K. Cognitive decline and mortality in a community-based sample of homeless and precariously housed adults: 9-year prospective study. BJPsych Open. (2020) 6:e21. 10.1192/bjo.2020.3 32043436 PMC7176832

[15] Mahmood Z Vella L Maye JE Keller AV Van Patten R Clark JMR. Rates of cognitive and functional impairments among sheltered adults experiencing homelessness. Psychiatr Serv. (2021) 72:333–7. 10.1176/appi.ps.202000065 33397143 PMC7952028

[16] Rogoz A Burke D. Older people experiencing homelessness show marked impairment on tests of frontal lobe function. Int J Geriatr Psychiatry. (2016) 31:240–6. 10.1002/gps.4316 26032583

[17] Gicas KM Jones AA Panenka WJ Giesbrecht C Lang DJ Vila-Rodriguez F. Cognitive profiles and associated structural brain networks in a multimorbid sample of marginalized adults. PLoS ONE. (2019) 14:e0218201. 10.1371/journal.pone.0218201 31194834 PMC6564539

[18] Weiser SD Hatcher A Frongillo EA Guzman D Riley ED Bangsberg DR. Food insecurity is associated with greater acute care utilization among HIV-infected homeless and marginally housed individuals in San Francisco. J Gen Intern Med. (2013) 28:91–8. 10.1007/s11606-012-2176-4 22903407 PMC3539018

[19] Burnam MA Koegel P. Methodology for Obtaining a Representative Sample of Homeless Persons: The Los Angeles Skid Row Study Evaluation Review. (1988). div data-widget-def= “general-html” data-widget-id= “07461a98-bd3b-44ba-95d5-ed4f187865c4” style=" color: rgb(51, 51, 51); font-family: arial; font-size: 14px; background-color: rgb(255, 255, 255).

[20] Kushel MB Perry S Bangsberg D Clark R Moss AR. Emergency department use among the homeless and marginally housed: results from a community-based study. Am J Public Health. (2002) 92:778–84. 10.2105/AJPH.92.5.778 11988447 PMC1447161

[21] Dunn LB Jeste DV. Enhancing informed consent for research and treatment. Neuropsychopharmacology. (2001) 24:595–607. 10.1016/S0893-133X(00)00218-9 11331139

[22] Beattie BL. Consent in Alzheimer's disease research: risk/benefit factors. Can J Neurol Sci. (2007) 34 Suppl 1:S27–31. 10.1017/S0317167100005527 17469678

[23] Sacco G Noublanche F Blazek F Hue C Carballido L Asfar M. How to deal with the consent of adults with cognitive impairment involved in European geriatric living labs? Philos Ethics Humanit Med. (2021) 16:3. 10.1186/s13010-021-00101-1 34130730 PMC8207703

[24] Sudore RL Schillinger D. Interventions to Improve Care for Patients with Limited Health Literacy. J Clin Outcomes Manag. (2009) 16:20–9. 20046798 PMC2799039

[25] Katz S Ford AB Moskowitz RW Jackson BA Jaffe MW. Studies of illness in the aged. The index of adl: a standardized measure of biological and psychosocial function. JAMA. (1963). 185:914–9. 10.1017/s0317167100005527 14044222

[26] Sullivan G Dumenci L Burnam A Koegel P. Validation of the brief instrumental functioning scale in a homeless population. Psychiatr Serv. (2001) 52:1097–9. 10.1176/appi.ps.52.8.1097 11474058

[27] Brown RT Hemati K Riley ED Lee CT Ponath C Tieu L. Geriatric conditions in a population-based sample of older homeless adults. Gerontologist. (2017) 57:757–66. 10.1093/geront/gnw011 26920935 PMC5881727

[28] Barry KL Fleming MF. The Alcohol Use Disorders Identification Test (AUDIT) and the SMAST-13: predictive validity in a rural primary care sample. Alcohol Alcohol. (1993) 28:33–42. 8471085

[29] Humeniuk R Ali R Babor TF Farrell M Formigoni ML Jittiwutikarn J. Validation of the alcohol, smoking and substance involvement screening test (ASSIST). Addiction. (2008) 103:1039–47. 10.1111/j.1360-0443.2007.02114.x 18373724

[30] Humeniuk R Newcombe DAL Dennington V Ali R. A randomised controlled trial of a brief intervention for illicit drug use linked to ASSIST screening in a primary healthcare setting: results from the Australian component of the World Health Organization Phase III ASSIST studies. Aust J Prim Health. (2018) 24:149–54. 10.1071/PY17056 29481765

[31] Radloff L. The CES-D scale: a self-report depression scale for research in the general population. Appl. Psychol. Measurement (1997). p. 385–401. 10.1177/014662167700100306

[32] van der Flier WM Scheltens P. Epidemiology and risk factors of dementia. J Neurol Neurosurg Psychiatry. (2005) 76 Suppl 5:v2–7. 10.1136/jnnp.2005.082867 16291918 PMC1765715

[33] Miebach L Wolfsgruber S Polcher A Peters O Menne F Luther K. Which features of subjective cognitive decline are related to amyloid pathology? Findings from the DELCODE study. Alzheimers Res Ther. (2019) 11:66. 10.1186/s13195-019-0515-y 31366409 PMC6668160

[34] Morley JF Cohen A Silveira-Moriyama L Lees AJ Williams DR Katzenschlager R. Optimizing olfactory testing for the diagnosis of Parkinson's disease: item analysis of the university of Pennsylvania smell identification test. NPJ Parkinsons Dis. (2018) 4:2. 10.1038/s41531-017-0039-8 29354684 PMC5768805

[35] Beach TG Adler CH Zhang N Serrano GE Sue LI Driver-Dunckley E. Severe hyposmia distinguishes neuropathologically confirmed dementia with Lewy bodies from Alzheimer's disease dementia. PLoS One. (2020) 15:e0231720. 10.1371/journal.pone.0231720 32320406 PMC7176090

[36] UCSF. TabCAT tasks. San Francisco: UCSF. (2021). 10.1038/385401a0

[37] Possin KL Moskowitz T Erlhoff SJ Rogers KM Johnson ET Steele NZR. The Brain Health Assessment for Detecting and Diagnosing Neurocognitive Disorders. J Am Geriatr Soc. (2018) 66:150–6. 10.1111/jgs.15208 29355911 PMC5889617

[38] Tsoy E Strom A Iaccarino L Erlhoff SJ Goode CA Rodriguez AM. Detecting Alzheimer's disease biomarkers with a brief tablet-based cognitive battery: sensitivity to Aβ and tau PET. Alzheimers Res Ther. (2021) 13:36. 10.1186/s13195-021-00776-w 33557905 PMC7871372

[39] UCSF. The EXAMINER Test Battery. San Francisco2021.

[40] Kramer JH Mungas D Possin KL Rankin KP Boxer AL Rosen HJ. NIH EXAMINER: conceptualization and development of an executive function battery. J Int Neuropsychol Soc. (2014) 20:11–9. 10.1017/S1355617713001094 24103232 PMC4474183

[41] Stiver J Staffaroni AM Walters SM You MY Casaletto KB Erlhoff SJ. The rapid naming test: Development and initial validation in typically aging adults. Clin Neuropsychol. (2021) 1–22. 10.1080/13854046.2021.1900399. [Epub ahead of print]. 33771087 PMC8464629

[42] Center MaA,. TabCat Tasks San Francisco: UCSF. (2019). Available online at: https://memory.ucsf.edu/research-trials/professional/tabcat/tabcat-tasks

[43] Jessen F Amariglio RE Buckley RF van der Flier WM Han Y Molinuevo JL. The characterisation of subjective cognitive decline. Lancet Neurol. (2020) 19:271–8. 10.1016/S1474-4422(19)30368-0 31958406 PMC7062546

[44] Sachs-Ericsson N Blazer DG. The new DSM-5 diagnosis of mild neurocognitive disorder and its relation to research in mild cognitive impairment. Aging Ment Health. (2015) 19:2–12. 10.1080/13607863.2014.920303 24914889

[45] Sachdev PS Blacker D Blazer DG Ganguli M Jeste DV Paulsen JS. Classifying neurocognitive disorders: the DSM-5 approach. Nat Rev Neurol. (2014) 10:634–42. 10.1038/nrneurol.2014.181 25266297

[46] Petersen RC. Mild cognitive impairment as a diagnostic entity. J Intern Med. (2004) 256:183–94. 10.1111/j.1365-2796.2004.01388.x 15324362

[47] McKeith IG Ferman TJ Thomas AJ Blanc F Boeve BF Fujishiro H. Research criteria for the diagnosis of prodromal dementia with Lewy bodies. Neurology. (2020) 94:743–55. 10.1212/WNL.0000000000009323 32241955 PMC7274845

[48] McKeith IG Boeve BF Dickson DW Halliday G Taylor JP Weintraub D. Diagnosis and management of dementia with Lewy bodies: Fourth consensus report of the DLB Consortium. Neurology. (2017) 89:88–100. 10.1212/WNL.0000000000004058 28592453 PMC5496518

[49] Rascovsky K Hodges JR Knopman D Mendez MF Kramer JH Neuhaus J. Sensitivity of revised diagnostic criteria for the behavioural variant of frontotemporal dementia. Brain. (2011) 134(Pt 9):2456–77. 10.1093/brain/awr179 21810890 PMC3170532

[50] McKhann GM Knopman DS Chertkow H Hyman BT Jack CR Kawas CH. The diagnosis of dementia due to Alzheimer's disease: recommendations from the National Institute on Aging-Alzheimer's Association workgroups on diagnostic guidelines for Alzheimer's disease. Alzheimers Dement. (2011) 7:263–9. 10.1016/j.jalz.2011.03.005 21514250 PMC3312024

[51] Armstrong MJ Litvan I Lang AE Bak TH Bhatia KP Borroni B. Criteria for the diagnosis of corticobasal degeneration. Neurology. (2013) 80:496–503. 10.1212/WNL.0b013e31827f0fd1 23359374 PMC3590050

[52] Gilman S Wenning GK Low PA Brooks DJ Mathias CJ Trojanowski JQ. Second consensus statement on the diagnosis of multiple system atrophy. Neurology. (2008) 71:670–6. 10.1212/01.wnl.0000324625.00404.15 18725592 PMC2676993

[53] Crutch SJ Schott JM Rabinovici GD Murray M Snowden JS van der Flier WM. Consensus classification of posterior cortical atrophy. Alzheimers Dement. (2017) 13:870–84. 10.1016/j.jalz.2017.01.014 28259709 PMC5788455

[54] Gorno-Tempini ML Hillis AE Weintraub S Kertesz A Mendez M Cappa SF. Classification of primary progressive aphasia and its variants. Neurology. (2011) 76:1006–14. 10.1212/WNL.0b013e31821103e6 21325651 PMC3059138

[55] Höglinger GU Respondek G Stamelou M Kurz C Josephs KA Lang AE. Clinical diagnosis of progressive supranuclear palsy: The movement disorder society criteria. Mov Disord. (2017) 32:853–64. 10.1002/mds.26987 28467028 PMC5516529

[56] Postuma RB Berg D Stern M Poewe W Olanow CW Oertel W. MDS clinical diagnostic criteria for Parkinson's disease. Mov Disord. (2015) 30:1591–601. 10.1002/mds.26424 26474316

[57] Prevention CfDCa. Diabetes (2022).

[58] Prevention CfDCa. High Cholesterol (2022).

[59] Prevention CfDCa. Hypertension (2022).

[60] Administration SAaMHS. Key Substance Use and Mental Health Indicators in the United States: Results From the National Survey on Drug Use and Health (2020).

[61] Prevention CfDCa. Traumatic Brain Injury (2022).

[62] Golimstok A Cámpora N Rojas JI Fernandez MC Elizondo C Soriano E. Cardiovascular risk factors and frontotemporal dementia: a case-control study. Transl Neurodegener. (2014) 3:13. 10.1186/2047-9158-3-13 24995127 PMC4080770

[63] Rasmussen Eid H Rosness TA Bosnes O Salvesen Ø Knutli M Stordal E. Smoking and obesity as risk factors in frontotemporal dementia and Alzheimer's Disease: the HUNT study. Dement Geriatr Cogn Dis Extra. (2019) 9:1–10. 10.1159/000495607 30792733 PMC6381906

[64] Wang HK Lee YC Huang CY Liliang PC Lu K Chen HJ. Traumatic brain injury causes frontotemporal dementia and TDP-43 proteolysis. Neuroscience. (2015) 300:94–103. 10.1016/j.neuroscience.2015.05.013 25982564

[65] Health NMHIo. Major Depression. (2019).

[66] Lyketsos CG Carrillo MC Ryan JM Khachaturian AS Trzepacz P Amatniek J. Neuropsychiatric symptoms in Alzheimer's disease. Alzheimers Dement. (2011) 7:532–9. 10.1016/j.jalz.2011.05.2410 21889116 PMC3299979

[67] Bang J Spina S Miller BL. Frontotemporal dementia. Lancet. (2015) 386:1672–82. 10.1016/S0140-6736(15)00461-4 26595641 PMC5970949

[68] Seeley WW. Anterior insula degeneration in frontotemporal dementia. Brain Struct Funct. (2010) 214:465–75. 10.1007/s00429-010-0263-z 20512369 PMC2886907

[69] Papinutto N Galantucci S Mandelli ML Gesierich B Jovicich J Caverzasi E. Structural connectivity of the human anterior temporal lobe: A diffusion magnetic resonance imaging study. Hum Brain Mapp. (2016) 37:2210–22. 10.1002/hbm.23167 26945805 PMC4922800

[70] Cadet JL Bisagno V Milroy CM. Neuropathology of substance use disorders. Acta Neuropathol. (2014) 127:91–107. 10.1007/s00401-013-1221-7 24292887 PMC7453825

[71] Idiaquez J Roman GC. Autonomic dysfunction in neurodegenerative dementias. J Neurol Sci. (2011) 305:22–7. 10.1016/j.jns.2011.02.033 21440258

[72] Rabinovici GD Miller BL. Frontotemporal lobar degeneration: epidemiology, pathophysiology, diagnosis and management. CNS Drugs. (2010) 24:375–98. 10.2165/11533100-000000000-00000 20369906 PMC2916644

[73] Rowe JB. Parkinsonism in frontotemporal dementias. Int Rev Neurobiol. (2019) 149:249–75. 10.1016/bs.irn.2019.10.012 31779815

[74] Snowden MB Bowen JD Hughes J Larson EB. Study of Alzheimer's dementia patients with parkinsonian features. J Geriatr Psychiatry Neurol. (1995) 8:154–8. 10.1177/089198879500800302 7576038

[75] Mendez MF Shapira JS. Loss of insight and functional neuroimaging in frontotemporal dementia. J Neuropsychiatry Clin Neurosci. (2005) 17:413–6. 10.1176/jnp.17.3.413 16179666

